# Supplementary material for: Evaluation of drug therapy problems, medication adherence and treatment satisfaction among heart failure patients on follow-up at a tertiary care hospital in Ethiopia
Source: PLoS One. 2020 Aug 28;15(8):e0237781. doi: 10.1371/journal.pone.0237781 (PMC7454938; doi:10.1371/journal.pone.0237781)
Supplement: S1 Questionnaire — (DOCX) [file pone.0237781.s001.docx]

## Annex I: Data abstraction format from medical chart and patient interviews

**Part I. Patients socio-demographic characteristics (Use “X” in the Boxes)**

| Card no | Age | Gender  Male Female | Pregnancy  Yes No |
| --- | --- | --- | --- |
| Marital status | Single | Married | Divorced widowed |
| Educational status | No formal edu. | Primary | Second. Tertiary |
| Place of residence |  |  |  |
| Social drug use | Cigarette smoking  Yes No | Alcohol use  Yes No | Khat chewing  Yes No |
| Salt restriction | Yes No |  |  |

**Part II Clinical characteristics (supplementary to the information obtained from medical chart)**

| NO |  |  |
| --- | --- | --- |
| 1 | Duration of heart failure treatment |  |
| 2 | Frequency of follow up |  |
| 3 | Comorbidity | Yes No |
| 4 | Total number of drug you are taking |  |
| 5 | How do you get your medications | Free Payment |

**Part-III:** Morisky Green Levin Medication Adherence Scale(MGL)

| **No** | **Items** | **No** | **Yes** |
| --- | --- | --- | --- |
| **1** | Do you ever forget to take your medicine? | €1 | €0 |
| **2** | Are you careless at times about taking your medicine? | €1 | €0 |
| **3** | When you feel better do you sometimes stop taking your medicine? | €1 | €0 |
| **4** | Sometimes you feel worse, when you take the medicine, do you stop taking it? | €1 | €0 |
|  | **Total score** |  |  |

If you have any problems that challenges your medication adherence please the select your reason (more than one answer is possible)

Patient forgets to take

Disbelief in drug effectiveness

 Fear of adverse events

Drug product too expensive

Drug product not available

Patient felt worse

Regimen complexity

**Part IV Assessment of Treatment satisfaction with medicine (SATMED-Q)**

For each question, **check** the number that best represents your opinion. There is no right or wrong answers. If you are not sure of any of the answers, check the one you consider most appropriate.

|  |  | **0** | 1 | **2** | | 3 | **4** |
| --- | --- | --- | --- | --- | --- | --- | --- |
| **1** | The side effects of the medicine interfere with my physical activities. |  |  |  | |  |  |
| **2** | The side effects of the medicine interfere with my leisure and free time activities. |  |  |  |  | |  |
| **3** | The side effects of the medicine interfere with my daily activities. |  |  |  |  | |  |
| **4** | 1. The medicine I am taking relieves my symptoms. |  |  |  |  | |  |
| **5** | I am satisfied with the time it takes for the medicine to start to work |  |  |  |  | |  |
| **6** | I feel better now than I did before starting the treatment. |  |  |  |  | |  |
| **7** | I find that taking my medicine is practical for me. |  |  |  |  | |  |
| **8** | I find it easy to use/take the medicine in its present form (taste, size, etc.). |  |  |  |  | |  |
| **9** | The timetable for taking the medicine suits me. |  |  |  |  | |  |
| **10** | Thanks to the medicine I am taking, it is easier for me to do my leisure and free time activities. |  |  |  |  | |  |
| **11** | Thanks to my medicine, it is easier for me to take care of my personal hygiene |  |  |  |  | |  |
| **12** | Thanks to my medicine, it is easier for me to perform my daily activities. |  |  |  |  | |  |
| **13** | My doctor has informed me in detail about my medical condition |  |  |  |  | |  |
| **14** | My doctor has informed me about the right way to treat my medical condition. |  |  |  |  | |  |
| **15** | I intend to continue using this treatment. |  |  |  |  | |  |
| **16** | I feel comfortable with my treatment. |  |  |  |  | |  |
| **17** | In general, I feel satisfied with the treatment |  |  |  |  | |  |

0 Not at all 1 A little bit 2 Somewhat 3 Quite a bit 4 Very muc

## Annex II: Data abstraction format from patient medical chart

Card Number________________ Age (in year) ____Weight (kg) _____Height (cm) ________

Present compliant:

| **No** | **Clinical characteristics** | | | | | |
| --- | --- | --- | --- | --- | --- | --- |
| 1 | Duration of heart failure |  | | | | |
| 2 | duration of heart failure medication |  | | | | |
| 3 | Frequency of follow up |  | | | | |
| 4 | NYHA class | Class 1 | | |  |  |
|  |  | Class 2 | | |  |  |
|  |  | Class 3 | | |  |  |
|  |  | Class 4 | | |  |  |
| 5 | Known drug allergy | No yes (specify) | | | | |
| 6 | Comorbidity  Yes No | **Type of comorbidity** | | **Etiology of heart failure** | | |
|  |  | HTN | MI | CRVHD | | |
|  |  | DM | Angina | Hypertensive heart disease | | |
|  |  | Dyslipidemia | Arrhythmia | Ischemic heart disease | | |
|  |  | AF |  | Cardiomyopathy | | |
|  |  | Stroke |  | PMI | | |
|  |  | PAD |  |  | | |
|  |  | DVT |  |  | | |

7 Past medical conditions and medications

| Medical condition/ Indication | Drug product (Generic Name) | Dosage regimen (dose, route, frequency, duration) | Date (dd/mm/yy) | | Response Effectiveness/ safety profile |
| --- | --- | --- | --- | --- | --- |
|  |  |  | Started | Stopped |  |
|  |  |  |  |  |  |
|  |  |  |  |  |  |
|  |  |  |  |  |  |
|  |  |  |  |  |  |
|  |  |  |  |  |  |
|  |  |  |  |  |  |
|  |  |  |  |  |  |

8 Physical Examination (PE)/vital signs: Consecutive record of visits

| **P/E** | **Date** |  |  |  |  |  |  |  |  |  |  |  |  |
| --- | --- | --- | --- | --- | --- | --- | --- | --- | --- | --- | --- | --- | --- |
| PR |  |  | | |  | | |  |  |  |  |  |  |
| BP |  |  | | |  | | |  |  |  |  |  |  |

9. Physical Examination(PE)/**vital** signs: Consecutive record of visits

| **Parameters** | Date(dd/mm/yy |  |  | |  | |  |  |  |  |  |  |  |  |  |
| --- | --- | --- | --- | --- | --- | --- | --- | --- | --- | --- | --- | --- | --- | --- | --- |
| Lipid profiles | LDL: mg/dl |  | | | | |  | | |  | | |  | | |
|  | TG: mg/dl |  | | | | |  | | |  | | |  | | |
|  | HDL: mg/dl |  | | | | |  | | |  | | |  | | |
|  | Total cholesterol |  | | | | |  | | |  | | |  | | |
| LFT | Date |  | |  | |  |  |  |  |  |  |  |  |  |  |
|  | SGPT (ALT |  | | | | |  | | |  | | |  | | |
|  | SGOT(AST) |  | | | | |  | | |  | | |  | | |
|  | ALP |  | | | | |  | | |  | | |  | | |
| RFT | Date |  | |  | |  |  |  |  |  |  |  |  |  |  |
|  | BUN |  | | | | |  | | |  | | |  | | |
|  | SrCr |  | | | | |  | | |  | | |  | | |
|  | GFR |  | | | | |  | | |  | | |  | | |
| Blood glucose | Date |  | |  | |  |  |  |  |  |  |  |  |  |  |
|  | FBS |  | | | | |  | | |  | | |  | | |
|  | RBS |  | | | | |  | | |  | | |  | | |
|  | HbA1C |  | | | | |  | | |  | | |  | | |
| Electrolytes | Date |  | |  | |  |  |  |  |  |  |  |  |  |  |
|  | Na |  | | | | |  | | |  | | |  | | |
|  | K |  | | | | |  | | |  | | |  | | |
|  | Mg |  | | | | |  | | |  | | |  | | |
|  | Ca |  | | | | |  | | |  | | |  | | |
|  | Cl |  | | | | |  | | |  | | |  | | |
|  | Date |  | |  | |  |  |  |  |  |  |  |  |  |  |
| CBC | WBC |  | | | | |  | | |  | | |  | | |
|  | RBC |  | | | | |  | | |  | | |  | | |
|  | Hgb |  | | | | |  | | |  | | |  | | |
|  | Hct |  | | | | |  | | |  | | |  | | |
|  | MCV |  | | | | |  | | |  | | |  | | |
|  | MCH |  | | | | |  | | |  | | |  | | |
|  | MCHC |  | | | | |  | | |  | | |  | | |
|  | PLT |  | | | | |  | | |  | | |  | | |
|  | PT |  | | | | |  | | |  | | |  | | |
|  | PTT |  | | | | |  | | |  | | |  | | |
|  | aPTT |  | | | | |  | | |  | | |  | | |
|  | INR |  | | | | |  | | |  | | |  | | |
| Other | Echo  ECG  MRI |  | | | | |  | | |  | | |  | | |

10 Present medical condition and medication

| Medical condition/ Indication | Drug product (Generic Name) | Dosage regimen (dose, route, frequency, duration) | Date (dd/mm/yy) | | Response /safety |
| --- | --- | --- | --- | --- | --- |
|  |  |  | Started | Stopped |  |
|  |  |  |  |  |  |
|  |  |  |  |  |  |
|  |  |  |  |  |  |
|  |  |  |  |  |  |
|  |  |  |  |  |  |
|  |  |  |  |  |  |
|  |  |  |  |  |  |
|  |  |  |  |  |  |

11 **Assessment of adverse drug reaction**

Was there any experienced adverse effect of the drugs? Yes No

If yes which of the following manifestation occur?

| Bleeding | Dry cough | Ankle edema | UGIB |
| --- | --- | --- | --- |
| Penicillin allergy | Hyperkalemia | angioedema | Gynecomastia |
| Headache | Bradycardia | Bronchospasm | GI |
| Unsafe drug |  |  |  |

Please specify the drug product and time of event

| Drug regimen | Adverse drug event | Date the event occurred |
| --- | --- | --- |
|  |  |  |
|  |  |  |
|  |  |  |

12 Is there any drug interaction Yes (specify) No

## Annex III: Modified DRPs Registration Format

| **DTPs Categories** | **Common Cause(s) of Drug therapy problem** |
| --- | --- |
| 1. Unnecessary drug therapy | - No medical indication - Non drug therapy more appropriate - Others ,specify______________________________ |
| 1. Needs additional drug therapy | - Untreated medical condition - Preventive/ prophylactic - Synergistic/ potentiating - Others, specify____________________________ |
| 1. Ineffective drug product | - More effective alternative is available - Condition refractory to drug - Dosage form inappropriate - Not effective for condition - Others, specify______________________________ |
| 1. Dose too low | - Wrong dose - Duration too short - Others, specify_______________________________ |
| 1. Adverse drug reaction | - Undesired effect - Unsafe drug for patient - Drug interaction - Dosage administered or changed too rapidly - Allergic reactions - Contraindications present - Others, specify_____________________________ |
| 1. Dose too high | - Wrong dose - Duration too long - Others, specify____________________________ |

# Annex IV Key informant interview for physicians

**Title:** Assessment of drug therapy problems among ambulatory heart failure patients attending at adult cardiac clinic of TASH

**Aim of the study:** to assess drug therapy problem and medication adherence and associated factors among heart failure patients at adult cardiac clinic of TASH.

* The following questions are based on the results obtained from drug therapy problem among heart failure patients at adult cardiac clinic of TASH. Please respond by briefly stating your opinion. Your response on these questions will make possible for the identification of the reasons behind some of the drug therapy problem encountered in heart failure patients.

Please put your signature based on your Qualification

R1

R2

R3

Thank you

1. In this study it was found that spironolactone was prescribed for almost all CRVHD patient who took furosemide, in your opinion what are the reasons to use spironolactone?
2. To prevent hypokalemia when the dose of furosemide is ≥ 40mg
3. To prevent hypokalemia when the dose of furosemide is ≥ 20mg
4. For its remodeling effect
5. Other(specify)
6. In this study patients who are allergic to penicillin didn’t receive alternative prophylaxis what do you think the reason could be?
7. In this study it was found that dose of ACEIs and B blockers are not being titrated in patients with left ventricular systolic dysfunction. What do you think the reason and at what interval do you consider dose titration?
8. In patients with HHD when do you think ASA and statins should be initiated
9. What do you think the reason behind use of ASA instead of warfarin in patients with AF
10. In heart failure patients with reduced ejection fraction Metoprolol tartrate and atenolol was indicated what could be the possible reasons
11. Which one of the guideline for treatment of heart failure are you using?
12. AHA/ACC B. ESC C. Ethiopian national guideline for NCDs
