# Supplementary material for: Evaluation of drug therapy problems, medication adherence and treatment satisfaction among heart failure patients on follow-up at a tertiary care hospital in Ethiopia
Source: PLoS One. 2020 Aug 28;15(8):e0237781. doi: 10.1371/journal.pone.0237781 (PMC7454938; doi:10.1371/journal.pone.0237781)
Supplement: S1 Rawdata — (DOCX) [file pone.0237781.s002.docx]

| **VARIABLES** | | | | | | **Total** | | **DTP (n=291)** | **No DTP (n=132)** |  |  |
| --- | --- | --- | --- | --- | --- | --- | --- | --- | --- | --- | --- |
| **Age Category** | | | | | | | | | | |  |
| 14-35 | | | | | | 111 | | 28 | 83 |  |  |
| 36-60 | | | | | | 214 | | 178 | 36 |  |  |
| >60 | | | | | | 98 | | 85 | 13 |  |  |
| **Sex** | | | | | | | | | | |  |
| Males | | | | | | 200 | | 115 | 85 |  |  |
| Female | | | | | | 223 | | 176 | 47 |  |  |
| **Marital status category** | | | | | | | | | | |  |
| Single | | | | | | 105 | | 59 | 46 |  |  |
| Married | | | | | | 270 | | 193 | 77 |  |  |
| Divorced | | | | | | 17 | | 14 | 3 |  |  |
| Widowed | | | | | | 31 | | 25 | 6 |  |  |
| **Educational status** | | | | | | | | | | |  |
| No formal education | | | | | | 133 | | 107 | 26 |  |  |
| Primary | | | | | | 126 | | 78 | 48 |  |  |
| Secondary | | | | | | 120 | | 75 | 45 |  |  |
| Diploma and above | | | | | | 44 | | 31 | 13 |  |  |
| **Social drug use** | | | | | | | | | | |  |
| Cigarette smoking | | | | | | Yes | 3 | 3 | - |  |  |
|  |  |  |  |  |  | No | 420 | 288 | 132 |  |  |
| Alcohol use | | | | | | Yes | 21 | 18 | 3 |  |  |
|  |  |  |  |  |  | No | 402 | 273 | 129 |  |  |
| Khat chewing | | | | | | Yes | 14 | 9 | 5 |  |  |
|  |  |  |  |  |  | No | 409 | 282 | 127 |  |  |
| **Source of medication** | | | | | | | | | | | |
| Free | | | | | | 227 | | 147 | 80 |  |  |
| Paid | | | | | | 196 | | 144 | 52 |  |  |
| **Hospitalization in the past one year** | | | | | | | | | | |  |
|  | | | | | | 156 | | 117 | 39 |  |  |
| **Cause of heart failure** | | | | | | | | | | |  |
| CRVHD | | |  | | | 215 | | 126 | 89 |  |  |
|  |  |  | MR | | | 51 | | 23 | 28 |  |  |
|  |  |  | TR | | | 28 | | 4 | 24 |  |  |
|  |  |  | AR | | | 24 | | 5 | 19 |  |  |
|  |  |  | AS | | | 5 | | 3 | 2 |  |  |
|  |  |  | MSMR | | | 48 | | 48 | - |  |  |
|  |  |  | MSAR | | | 28 | | 27 | 1 |  |  |
|  |  |  | MRAR | | | 15 | | 3 | 12 |  |  |
|  |  |  | MRAS | | | 11 | | 11 | - |  |  |
|  |  |  | MSAS | | | 5 | | 5 | - |  |  |
| IHD | | | | | | 91 | | 78 | 13 |  |  |
| HHD | | | | | | 62 | | 37 | 25 |  |  |
| DCMP | | | | | | 45 | | 43 | 2 |  |  |
| Others | | | | | | 10 | | 7 | 3 |  |  |
| **Duration of heart failure treatment** | | | | | | | | | | |  |
| ≤5 years | | | | | | 235 | | 128 | 107 |  |  |
| 6-10 years | | | | | | 135 | | 114 | 21 |  |  |
| >10 years | | | | | | 53 | | 49 | 4 |  |  |
| **NYHA class** | | | | | | | | | | |  |
| Class I | | | | | | 63 | | 15 | 48 |  |  |
| Class II | | | | | | 125 | | 61 | 64 |  |  |
| Class III | | | | | | 235 | | 215 | 20 |  |  |
| **LVEF** | | | | | | | | | | |  |
| HFrEF | | | | | | 104 | | 96 | 8 |  |  |
| HFmrEF | | | | | | 39 | | 25 | 14 |  |  |
| HFpEF | | | | | | 280 | | 170 | 110 |  |  |
| **Frequency of follow up** | | | | | | | | | | |  |
| Every month | | | | | | 77 | | 46 | 31 |  |  |
| Every 2 month | | | | | | 78 | | 50 | 28 |  |  |
| Every 3 month | | | | | | 161 | | 113 | 48 |  |  |
| Every 4 month | | | | | | 70 | | 55 | 15 |  |  |
| Every 6 month | | | | | | 37 | | 27 | 10 |  |  |
| **Systolic blood pressure** | | | | | | | | | | |  |
| <120 | | | | | | 237 | | 139 | 98 |  |  |
| 120-129 | | | | | | 153 | | 119 | 34 |  |  |
| 130-139 | | | | | | 25 | | 25 | - |  |  |
| ≥140 | | | | | | 8 | | 8 | - |  |  |
| **Comorbidities** | | | | | | | | | | |  |
| Hypertension | | | | | Yes | 173 | | 136 | 37 |  |  |
|  |  |  |  |  | No | 250 | | 155 | 95 |  |  |
| Atrial fibrillation | | | | | Yes | 158 | | 149 | 9 |  |  |
|  |  |  |  |  | No | 265 | | 142 | 123 |  |  |
| Diabetes | | | | | Yes | 67 | | 58 | 9 |  |  |
|  |  |  |  |  | No | 356 | | 233 | 123 |  |  |
| Dyslipidemia | | | | | Yes | 26 | | 20 | 6 |  |  |
|  |  |  |  |  | No | 397 | | 271 | 126 |  |  |
| Asthma | | | | | Yes | 21 | | 20 | 1 |  |  |
|  |  |  |  |  | No | 402 | | 271 | 131 |  |  |
| Stroke | | | | | Yes | 18 | | 17 | 1 |  |  |
|  | | | | | No | 405 | | 274 | 131 |  |  |
| Peripheral neuropathy | | | | | Yes | 11 | | 9 | 2 |  |  |
|  |  |  |  |  | No | 412 | | 282 | 130 |  |  |
| Others | | | | | Yes | 38 | | 32 | 6 |  |  |
| **Number of comorbidity** | | | | | | | | | | |  |
| One comorbidity | | | | | | 173 | | 149 | 24 |  |  |
| Two comorbidity | | | | | | 91 | | 76 | 15 |  |  |
| Three or more comorbidity | | | | | | 54 | | 48 | 6 |  |  |
| **Number of medication** | | | | | | | | | | |  |
| ≤ 5 | | | | | | 155 | | 47 | 108 |  |  |
| > 5 | | | | | | 268 | | 244 | 24 |  |  |
|  | | | | | |  | |  |  |  |  |
| **Pattern of drug therapy problems** | | | | | | | | | | |  |
|  | | | | **Total** | | **CRVHD** | | **IHD** | **HHD** | **CMP** | **Others** |
| No medical indication | | | | 17 | | - | | 14 | 2 | 1 |  |
| Non drug therapy | | | | 14 | | 13 | | - | - | - | 1 |
| Prophylactic/preventive | | | | 55 | | 15 | | 25 | 3 | 12 | - |
| Synergy | | | | 14 | | 7 | | - | 7 | - | - |
| Ineffective drug | | | | 100 | | 23 | | 50 | 5 | 18 | 4 |
| Dose low | | | | 52 | | 3 | | 29 | - | 20 | - |
| Dose high | | | | 4 | | 4 | | - | - | - | - |
| ADR | |  | | 84 | | 36 | | 22 | 21 | 4 | 1 |
|  | | Gum bleeding | | 22 | | 21 | | 1 | - | - | - |
|  |  | Dry cough | | 20 | | 2 | | 11 | 6 | 1 | - |
|  |  | Ankle edema | | 11 | | - | | 2 | 7 | 2 | - |
|  |  | Penicillin allergy | | 5 | | 5 | | - | - | - | - |
|  |  | Unsafe drug | | 5 | | - | | - | 5 | - | - |
|  |  | Others* | | 21 | | 8 | | 8 | 3 | - | 2 |
| Others: angioedma, bradycardia, bronchospasm, GI irritation,gynecomastia, hyperkalemia,hypotension, myopathy,UGIB, | | | | | | | | | | | |
| **DI** |  | | | 199 | | 95 | | 51 | 19 | 31 | 3 |
|  | One DI | | | 141 | | 78 | | 30 | 13 | 18 | 2 |
|  | Two DI | | | Two DI | | 42 | | 16 | 11 | 7 | 7 |
|  | Three and more | | | 16 | | 1 | | 9 | 1 | 5 | - |
| **Non adherence** | | | | 191 | | 85 | | 49 | 28 | 25 | 4 |
| **Possible reasons for non-adherence** | | | |  | | | | | | | |
| 1. Forgetfulness | | | | 184 | | 85 | | 49 | 27 | 19 | 4 |
| 2.Medication side effect | | | | 82 | | 45 | | 23 | 8 | 5 | 1 |
| 3. Regimen complexity | | | | 169 | | 78 | | 49 | 21 | 19 | 2 |
| 4. Drug unavailability | | | | 94 | | 41 | | 32 | 8 | 9 | 4 |
| 5. cost of medication | | | | 63 | | 15 | | 29 | 13 | 6 | - |
| 6. patient belief on drug effectiveness | | | | 83 | | 36 | | 23 | 20 | 3 | 1 |
