# Supplementary material for: Evaluation of drug therapy problems, medication adherence and treatment satisfaction among heart failure patients on follow-up at a tertiary care hospital in Ethiopia
Source: PLoS One. 2020 Aug 28;15(8):e0237781. doi: 10.1371/journal.pone.0237781 (PMC7454938; doi:10.1371/journal.pone.0237781)
Supplement: S1 File — (DOCX) [file pone.0237781.s003.docx]

| Definition of DTP |  |
| --- | --- |
| Drug Therapy Problem (DTP) | DTP is any undesirable event experienced by a patient which involves, or is suspected to involve, drug therapy, and that interferes with achieving the desired goals of therapy, which can be identified using European society of cardiology and the American heart association (AHA) heart failure guidelines. |
| Unnecessary drug therapy: | The patient is taking a medication for no medically valid indication |
| Non-drug therapy more appropriate: | The medical condition is most appropriately treated with non-drug therapy |
| More effective drug available | Patient is not on first-line treatment agent in accordance with published guidelines for a particular condition (with no documented contraindication to its use) |
| Prophylactic/preventive: | A drug therapy required to prevent development of new condition or to prevent disease progression |
| Synergistic /additive effect: | The patient requires additional or combination therapy to achieve treatment goal (especially for patients with hypertension) |
| Suboptimal dosing: | Medications are not titrated towards established target doses or towards highest tolerated dose after giving adequate time and considering individual patient condition. |
| Dosage high: | The dose is high enough to cause ADR |
| ADR: | Any noxious, unintended, and undesired effect associated with the drug experienced by the patient that is documented in the patient chart as self-reported by patients and interpreted as ADR by attending physicians. |
