# Supplementary material for: Evaluation of drug therapy problems, medication adherence and treatment satisfaction among heart failure patients on follow-up at a tertiary care hospital in Ethiopia
Source: PLoS One. 2020 Aug 28;15(8):e0237781. doi: 10.1371/journal.pone.0237781 (PMC7454938; doi:10.1371/journal.pone.0237781)
Supplement: Attachment — Submitted filename: Response to reviewer comments.docx [file pone.0237781.s004.docx]

# Reviewers’ Comments and Authors Response

Manuscript Number:  PONE-D-19-34755

**Manuscript Title:** Evaluation of Drug Therapy Problems, Medication Adherence and Treatment Satisfaction among Heart Failure Patients on Follow-up at a Tertiary Care Hospital in Ethiopia

**Authors:** Elham Seid, Ephrem Engidawork, Desalew Mekonnen, Minyahil Alebachew, Alemseged Beyene

We would like to thank the Editor and reviewers for their critical comments and constructive suggestions, which contributed to the quality of the manuscript. We have revised the manuscript as advised and changes made are highlighted in yellow. Point-by-point response for the reviewers’ comment is given below. We hope the revision undertaken has improved the manuscript to a level of your satisfaction and we request your editorial hand.

**Response to Editorial Comments**

**Comment 1:** Definition of DTP with supplementary table

**Response 1**: Accommodated.

**Comment 2:** Drug interaction as DTP since all top 5 drug interaction are clinically not relevant (hardly considered as DTP )

**Response 2:** Removed all the others except those associated with adverse drug reactions.

**Comment 3:** Clearly divide between HFrEF and HFmrEF

**Response 3:** Accommodated.

**Comment 4:** The discussion should be short and focused significantly

**Response 4:** Shortened as much as possible

**Comment 5:** Permission for MMA4

**Response 5:-** Since we did not hear from the License holder, we used the equivalent scale “the four –item Morisky Green Levin Medication Adherence Scale (MGL)” , which is a free online version.

**Comment 6:** Mention statistical analysis used

**Response 6:** We elaborated the statistical analysis used and also included in the abstract.

**Response to Reviewer' comments**

**Comment 1:** Please specify further the types of valve involved in CRVHD patients

**Response 1:** The types of valve lesion involved are provided in a new Figure (Figure 1). Please take note that patients with decompensated MS were not included in the study.

**Comment 2:** If HF results from VHD, then a class I indication exists for valvular intervention. Please specify the reasons why this has not been performed.

**Response 2:** Valvular surgery was not provided when the study was initiated. It used to done through mission based aid or sponsor based travel abroad.

**Comment 3:** Please specify the length of HF duration according to etiology of HF.

**Response 3:** Accommodated (See Table 4).

**Comment 4:** Generalizability is limited because of the relatively young age and high prevalence of CRVHD

**Response 4:** Dear reviewer: I agree with your point, however since diagnosis in developing countries like Ethiopia is usually established at an advanced stage of the disease, more than half of the patients are above 35 years old. Moreover, almost half of the patients included in this study had heart failure caused by other than CRVRHD.

**Comment 5:** A prevalence of HFpEF of 66.2% in their group. However, this will be enriched with CRVHD patients (probably MV stenosis).

**Response 5:** Dear reviewer: thank you for your valuable comment, but as it is indicated in specific valve involved; in this study patients with MS also have other valve lesions. Moreover, the classification of ejection fraction was taken from the patient’s medical chart.

**Comment 6:** Specify the reason for inappropriate dose (suboptimal dose)

**Response 6:** The dose low is considered as DTP if patients with HFrEF and on ACEIs or beta blockers were not up titrated after carefully consideration of patient status and after excluding any comorbidity or contraindication and the patient at high risk of toxicity due to increased dose (patients who had an organ dysfunction). Unfortunately, as I have raised in the discussion part patients remain in the same dose for several years ( for example patients with IHD and EF of 34 might be on Metoprolol 12.5 mg for years)

**Comment 7:** Anecdotic interactions are scored as DTPs (such as diuretic with aspirin; or spironolactone with aspirin etc

**Response 7:** We have made a correction and removed such types of drug interactions

**Comment 8:** Generalisablity is limited, because of the very high prevalence of untreated CRVHD and young patient age.

**Response 8:** As per the suggestion; we have made a correction in the discussion part. The main problem is there were only a few studies available and even these studies were done outside Africa, where the cause is not RHD. However we have tried to use these studies only if the problem is almost identical. For example patient with reduced EF and not on cardio protective therapy are considered to have DTP irrespective of the cause of heart failure.

**Comment 9:** Dual antiplatelet therapy is class I recommended in ACS patients. Therefore, this should not be regarded as a DDI.

**Response 9:** Accommodated.

**Comment 10:** Expand Table 4 with patient characteristics, such as age, comorbidities, gender, renal function (eGFR), blood pressure and medication

**Response 10:** We have made a correction as per recommendation in Table 3. Except eGFR as renal function test is not routinely performed in the outpatient setting.

**Comment 11:** How many patients have been hospitalized for HF in the past?

**Response 11:** Dear reviewer, I do have a data that showed only the number of hospitalized patients on the past one year. So I included the frequency (%) in Table 1.

**Comment 12:** Please have the manuscript reviewed by a native English speaker.

**Response12:** Accommodated.
